# Electrospun Poly(vinylpyrrolidone)/*Thymus vulgaris* L. Mats for the Protection of Fresh Berries Against Spoilage

**DOI:** 10.3390/ma19091874

**Published:** 2026-05-01

**Authors:** Erika Adomavičiūtė, Egidijus Griškonis, Visvaldas Varžinskas, Virginija Jankauskaitė

**Affiliations:** 1 Department of Production Engineering, Faculty of Mechanical Engineering and Design, Kaunas University of Technology, Studentu Str. 56, 51424 Kaunas, Lithuania; virginija.jankauskaite@ktu.lt; 2 Center for Research on Advanced Packaging Materials and Technologies, Faculty of Mechanical Engineering and Design, Kaunas University of Technology, Studentu Str. 56, 51424 Kaunas, Lithuania; visvaldas.varzinskas@ktu.lt; 3 Department of Physical and Inorganic Chemistry, Faculty of Chemical Technology, Kaunas University of Technology, Radvilenu pl. 19, 50254 Kaunas, Lithuania; egidijus.griskonis@ktu.lt

**Keywords:** food packaging, poly(vinylpyrrolidone), electrospinning, *Thymus vulgaris* L., citric acid

## Abstract

The use of non-biodegradable plastic food packaging materials has become a major environmental concern. These plastics release chemicals and microplastics during degradation, harming wildlife and entering the food chain, posing risks to both environmental and human health. This study aimed to evaluate electrospun poly(vinylpyrrolidone) (PVP) mats incorporating natural antibacterial *Thymus vulgaris* L. extract (TE) and natural crosslinker citric acid (CA) as alternative food packaging materials. Packaging mats with TE and/or CA combinations in PVP were evaluated for their structural, chemical, optical, and shelf-life-enhancing effects on blueberries. The results show that dissolving PVP in TE extract and adding CA in PVP ethanol-water or TE-based solutions significantly affected the viscosity and conductivity of the electrospinning solutions, thereby influencing the morphology of electrospun mats. FTIR analysis confirmed the incorporation of TE into the polymer and indicated CA induced hydrogen bonding, interactions that may reduce the polymer chain mobility and increase the brittleness of the electrospun mat. In tests with blueberries, it was estimated that the commonly used traditional food film minimized blueberry weight loss, whereas the porous electrospun PVP and PVP/TE mats allowed greater moisture release and preserved better visual quality by reducing wrinkling and dehydration. Overall, electrospun PVP-based mats functionalized with TE show promise as sustainable food packaging materials that balance moisture management with product appearance.

## 1. Introduction

Electrospinning is a simple and cost-effective method for forming nonwoven materials (mats) from nano-microfibers. Electrospun nonwoven mats have unique properties: small fiber diameter (in the nano range), controllable porosity, and high specific surface area. Due to these unique properties and the ability to use different types of polymers (synthetic, natural, biodegradable) electrospun materials have widely application areas: biomedical field (wound dressing, drug delivery system, scaffolds for tissue engineering, cosmetic face mask), filters, active or smart packaging materials, materials for sensors, etc.

Smart and active packaging materials are gaining increasing attention because they provide additional functionalities beyond traditional barrier properties. These include moisture absorption, antioxidant release, antibacterial activity, flavor or odor absorption, and color change indicators. Due to their unique characteristics, electrospun mats provide several advantages compared with traditional film packaging. Their porous structure and high responsiveness to environmental changes (e.g., relative humidity and temperature), as well as their ability to encapsulate antioxidants, antibacterial agents, freshness indicators, or gas sensors, make them highly promising for advanced packaging applications [1,2]. According to Yu and coauthors’ study [3], there are two main types of polymers used for electrospinning: natural and synthetic polymers. Among the natural polymers, chitosan (CS), gelatin, zein, cellulose, and collagen are usually used for food packaging as a result of their good biocompatibility, high number of reactive groups, and low cost. Synthetic polymers, such as polylactic acid (PLA), polyvinyl alcohol (PVA), polycaprolactone, and poly(vinylpyrrolidone) (PVP), are also widely used in electrospinning [3].

Electrospun mats with natural extract or essential oils have been actively investigated as materials for wound healing and as active, smart packaging materials. The key is to incorporate bioactive materials consisting of polyphenols in electrospun mats and ensure their stability. Polyphenols, abundant in plant foods such as fruits, vegetables, and tea, are bioactive compounds with antioxidant, anti-inflammatory, and antibacterial properties [4,5]. Edible electrospun mats with antioxidant and antimicrobial properties have been successfully developed from a wide range of polymer extract combinations, including PVA with sumac extract [5]; PVA with green tea extract for the shelf life of fresh kiwi [6]; octenylsuccinylated starch and pullulan with tea polyphenols as active packaging for fish fillets [7]; gelatin with green tea extract for beef shelf life preservation [8]; PCL/casein with essential oils of green tea [9], gelatin/zein with grape pomace extract for walnut protection [10]; PCL and chitosan (CS) with encapsulated curcumin nanoparticles for antimicrobial food packaging [3]; and PLA film coated with coaxial electrospun chitosan with clove and argan oils for biodegradable packaging materials with antimicrobial properties [11].

PVP (poly(N-vinyl-2-pyrrolidone) is widely used for medical, pharmaceutical, food, cosmetic, and other applications. It is made by free radical polymerization of N-vinyl-2-pyrrolidone and, like its monomer, is soluble in a large number of polar solvents, including water. The US Food and Drug Administration has approved PVP as a non-toxic safety [12,13,14,15]. In addition, PVP is a biodegradable synthetic polymer with excellent electrospinning ability. Y. Li and coauthors [16] loaded eugenol in PVP and formed edible electropun mats that effectively prolonged the shelf life of strawberries.

Thyme (*Thymus vulgaris* L., (*T. vulgaris*)), one of the family Lamiaceae, has been considered for antifungal and antioxidant activity owing to the phytochemicals’ ability in the extract. Thyme, due to its main active constituents, thymol and carvacrol, provides a prospective basis for fruitful bioactive agents with excellent properties such as antioxidant, antimicrobial, and anti-inflammatory capability [17,18,19,20,21,22]. Owing to these properties, it is widely used in medical applications and smart food packaging. H. Maleki and coauthors [17] electrospun a PVA/CS mat with thyme and ginger extracts (*Zingiber officinale*) for wound dressing. The electrospun mat showed high antioxidant activity, and its release profile was continuous and sustained for almost 72 h. It was observed that the release of TE had a lower diffusion rate from the PVA/CS mat and from ginger extract. The authors assumed this was due to the more hydrophobic compounds in the extract, e.g., thymol and carvacrol. E. C. Peixoto et al. [19] analyzed electrospun zein mats encapsulating *T. vulgaris* as essential oil (TEO) for meat packaging. Electrospun zein mats showed moderate antioxidant activity, which increased significantly against the ABTS radical when 30% TEO was added, reaching 98% inhibition. Higher levels of TEO (40–60%) reduced this effect due to weaker interactions between zein and TEO. Zein mats with 60% TEO showed antimicrobial activity against *E. coli* and *Staphylococcus* in meat packaging during seven days of refrigerated storage [19]. Covering the sweet potato starch film with a zein layer containing TEO enhances the bioactive and biodegradable properties of the packaging material compared with the plain starch film [18]. L.M. Fonseca and coauthors [20] encapsulated TEO in starch electrospun mats as a material for food packaging and proved that TEO is an excellent natural substitute for synthetic compounds to maintain the quality and safety of food products. Furthermore, thyme oleoresin (TER) was successfully incorporated into electrospun PLA mats [21]. The PLA/TER 15% composite exhibited strong antioxidant activity, effectively suppressing thiobarbituric acid reactive substance formation in meat samples. This indicates its potential to delay oxidative deterioration and extend the shelf life of meat products. TER is a viscous, herbaceous-scented liquid obtained by n-hexane extraction of dried *T. vulgaris* leaves. It is known for its potent antibacterial, anti-inflammatory, antifungal, and antioxidant properties, making it a promising natural additive for active food packaging applications [21]. TEO nanoemulsions was incorporated in electrospun PCL mats [22]. The electrospun PCL/TEO nanoemulsion mats showed antimicrobial activity against major foodborne pathogens *E. coli*, *S. enterica*, *S. aureus*, *B. cereus*. To improve the stability of natural preservatives, *N. shirdam* with coauthors [23] had fabricated and evaluated an electrospun double-layer mat. The inner layer of the electrospun mat was chitosan/PVA loaded with nisin and Shirazi TEO; the outer layer is cellulose acetate containing TEO. All these studies [18,19,20,21,22,23] demonstrated excellent properties of *T. vulgaris* extracts as a natural biologically active antibacterial and antioxidant material suitable for food packaging.

Carboxylic acids have been widely used as green crosslinkers that can impart water stability to electrospun mats. Among them, citric acid (CA) has been shown to effectively improve the properties of products developed from carbohydrates and proteins [24]. D. Nataraj et al. [24] had used CA as a crosslinking agent in PVA electrospun solution to evaluate the ability of the PVA mats to support the growth and proliferation of mouse fibroblasts. Research studies [25,26,27,28] have shown that crosslinked PVA/CA electrospun mats could be an excellent material to absorb moisture and deliver bioactive substances to diversify active food packaging applications or filters. The crosslinking process of PVA with CA had improved the thermal stability, mechanical properties, contact angle, and water stability of the electrospun mats [25]. The combination of clove oil [25] and oregano essential oil [26] incorporated into PVA mats cross-linked with CA demonstrated antibacterial properties. In addition to its well-established role as a covalent crosslinking agent for hydroxyl- and amine-containing biopolymers, citric acid has also been reported to influence the functional behavior of more chemically inert or weakly reactive polymer systems. Even in cases where direct covalent bond formation is limited, CA may contribute to network stabilization through secondary interactions such as hydrogen bonding, ionic interactions, and physical entanglement within multicomponent polymer matrices [29].

The aim of this study is to develop a water-soluble, environmentally sustainable active food packaging electrospun mat incorporating biologically active compounds, and to comprehensively evaluate its structural and chemical characteristics, as well as its effectiveness in preserving the postharvest quality of blueberries. Owing to its low toxicity, regulatory acceptance for food-contact applications, and multifunctional contribution to polymer network formation and stabilization, CA was selected as a green agent to improve the functional performance of the developed PVP system. In this context, CA acts not merely as a conventional cross-linker but as a versatile structural modifier capable of enhancing physicochemical performance, environmental safety, and functional versatility of polymer-based materials.

## 2. Materials and Methods

### 2.1. Materials

Polyvinylpyrrolidone (PVP) powder (Mw 1,300,000) was supplied by Sigma Aldrich (Darmstadt, Germany). TE was supplied from “Acorus Calamus” (Švenčionys, Lithuania), while citric acid (C_6_H_8_O_7_) powder was purchased from Sigma Aldrich (Germany). Ethanol (96 vol%) was used as a solvent for the production of *Thymus vulgaris* extract (TE).

### 2.2. Preparation of TE and Electrospun Solutions

The extract of TE was produced by using a solution of water–ethanol (1:9) for extraction of raw thyme and mixing with a magnetic stirrer IKA Werke Yellowline MSH Basic S (IKA-Werke GmbH & Co. KG, Staufen, Germany) for 6 h at room temperature. The ratio of raw thymus to extractive agent was 3:7. After mixing, TE was kept at room temperature for 24 h to allow swelling. TE was filtered through Whatman^®^ qualitative filter paper, Grade 1 (Cytiva, Marlborough, MA, USA). The ranges of the concentration of phenolic compounds were 27.45 ± 2.55 mg GAE/g, the flavonoids 19.05 ± 0.65 mg QE/g of TE was estimated at the Lithuanian Research Centre for Agriculture and Forestry.

A pure 10 wt% PVP solution was obtained by dissolving powder in water–ethanol mixture (1:9, *v*/*v*), whereas PVP/TE solution was prepared by dissolving PVP directly in the TE extract under magnetic stirring for 4 h. Different amount of CA, namely 4 wt% (4 CA) and 10 wt% (10 CA), were added to the prepared PVP-based solutions, followed by additional mixing for 4 h. The solutions were allowed to age for 24 h under ambient conditions prior to electrospinning.

### 2.3. Estimation of Viscosity and Electric Conductivity of Solutions

Viscosity of polymer solutions was determined by the viscosimeter “Brookfield DV-II + Pro” (Brookfield Engineering Laboratories USA, Middleboro, MA, USA). Conductivity of electrospun solution was estimated with HQ40D multiparameter meter (Hach, USA, Loveland, CO, USA). Before the measurements, each solution sample was equilibrated to a room temperature of 22 ± 2 °C to ensure uniform conditions across all specimens. The viscosity and electric conductivity of each sample was measured three times, and the average was calculated.

### 2.4. Electrospinning of PVP Mats

Nonwoven mats from nano-microfibers were formed using “Nanospider™” (Elmarco, Liberec, Czech Republic) electrospinning equipment. Electrospun mats from solution were formed at applied voltage *U* = 50 kV, distance between electrodes *L* = 13 cm, temperature of environment maintained at *T* = 23 ± 2 °C, and air humidity φ = 55 ± 4%. Time of electrospun mat formation (nano-microfibers collection)—5 min.

### 2.5. Estimation of the Structure of Electrospun Mats

The morphology and diameters of electrospun packaging mats were examined using a scanning electron microscope (SEM: S-3400N, Hitachi, Tokyo, Japan) operated at beam voltage of 3 kV and magnification of 50× (scale bar: 1 mm). Fiber diameters were measured from SEM images using NIS-Elements D software (version 5.x, Nikon Corporation, Tokyo, Japan). The average diameter was calculated based on measurements of 150 nano-microfibers.

### 2.6. FT-IR Analysis of Electrospun Mats

FT-IR Attenuated Total Reflectance (ATR) spectra were recorded using a Frontier spectrophotometer (Perkin Elmer, Waltham, MA, USA). A small amount of the sample was pressed against a diamond crystal plate, and the spectrum was recorded in the wavenumber range from 4000 to 560 cm^−1^. The number of scans was 6, and the resolution was 4 cm^−1^. The data were processed using the Spectrum 10 Spectroscopy Software (Perkin Elmer, USA).

### 2.7. Optical Properties

To investigate how the UV-VIS diffuse reflectance spectra of electrospun mats changed, they were measured with a Lambda 35 UV/VIS spectrometer (Perkin-Elmer, Waltham, MA, USA) in a wavelength range of 200–800 nm.

### 2.8. Berries Storage and Preservation Test

Twelve glass containers were prepared for the blueberry preservation experiment. Three containers were covered with food-grade low-density polyethylene (PE) wrapping film, three with electrospun PVP mats, three with PVP/TE mats, and three were left uncovered as the control group. All blueberries selected for the study were free from physical damage and fungal spoilage. The berries were divided into 12 groups, each containing nine berries, and distributed into the glass containers (three berries per container). The containers were stored under ambient conditions (*T* = 22 ± 3 °C, relative humidity *φ* = 58 ± 5%). Over a storage period of 96 h, the containers were periodically weighed, and their weight loss and visual quality of blueberries were assessed. Weight loss was expressed as the percentage reduction relative to the initial weight. For the assessment of berry quality, digital image analysis was employed to calculate the wrinkle area index (WAI) and the shrinkage index (SI), expressed as relative area. The WAI was used to objectively quantify surface texture irregularities and wrinkling of the berries. A normalized scale ranging from 0 to 1 was applied (0 represents a perfectly smooth surface and 1.0 corresponds to a fully desiccated and wrinkled surface). The SI was calculated as the ratio of the projected area of the berry at a given storage time to its initial projected area in the fresh, healthy state.

## 3. Results

### 3.1. The Effect of TE and CA on Viscosity and Conductivity of Solutions

The viscosity and electrical conductivity of different electrospun polymer solutions are shown in Table 1. Solution concentration is a critical parameter in optimizing electrospun mats’ morphology, as it directly influences the spinnability of the polymer solution. When the concentration is too low, inadequate chain entanglement results in the formation of droplets (electrospraying) rather than continuous nano-micro fibers [30]. The dissolution of PVP in TE increased the viscosity of the electrospinning solution by 34% compared with the water–ethanol PVP solution. The insertion of 4 wt% and 10 wt% CA in electrospinning solutions caused an increase in viscosity of 33% and 39% in the water–ethanol PVP solution and 35%, 54% in the PVP in TE extract, respectively. The increase in viscosity upon TE and the addition of CA may be attributed to the formation of hydrogen-bonded networks and intermolecular interactions between TE, CA, and PVP polymer chains. The same results were stated in the study [19]; the incorporation of TEO into the zein polymer solution increased the viscosity of the electrospun solutions, according to the authors, possibly due to the interaction of TEO with the zein polymer. Contrary results were reported regarding the viscosity of electrospun PVA solutions containing CA. In the study by R. Ren and coauthors [26], viscosity gradually decreased as the amount of CA in the PVA solution increased, which may be attributed to a dilution effect caused by the low molecular weight CA. The difference in viscosity trends may also be influenced by the fact that water was used as the solvent for PVA.

The conductivity reflects the charge density and the resulting electrostatic repulsion on the surface of the electrospun polymer jet, thereby determining the extent of its elongation. The conductivity of PVP solutions prepared with TE and/or CA increased more than 20-fold (Table 1). According to E.C. Peixoto and coauthors’ study [19], adding 30% TEO decreased electrical conductivity more than the essential-oil-free zein solution, whereas a solution containing 60% TEO exhibited the highest conductivity. This indicates that conductivity changes are strongly dependent on the type and concentration of bioactive additives, as well as on their interactions with the polymer matrix. Adding CA to PVA [25] led to an increase in the conductivity of PVA solutions due to the ionization of citric acid.

### 3.2. The Effect of TE and CA on Fiber Diameter of Electrospun Mats

**Figure 1:**
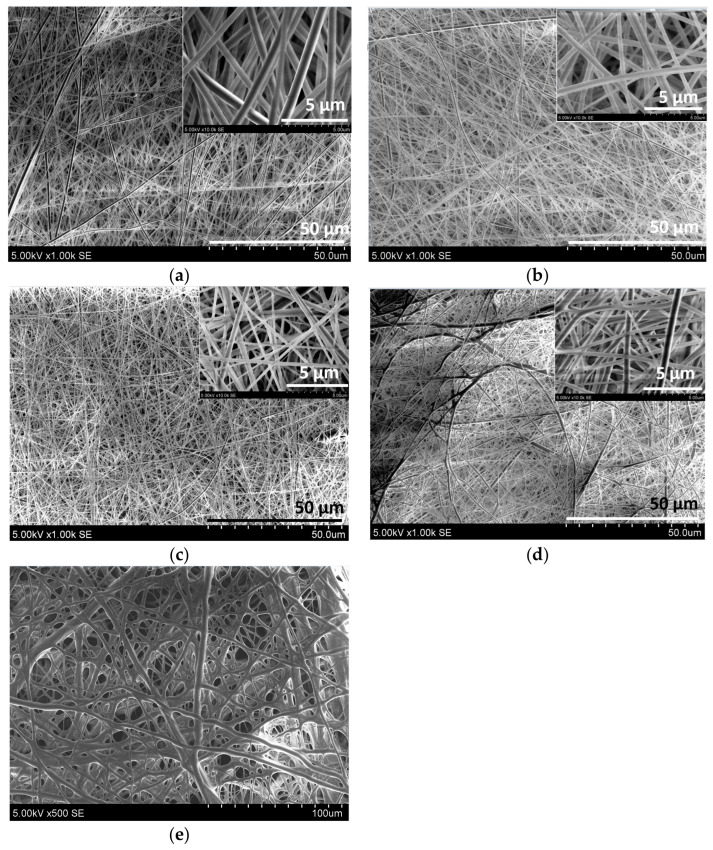
SEM images of PVP based electrospun mats: (**a**) PVP (1000×); (**b**) PVP/TE (1000×); (**c**) PVP + 4 CA (1000×); (**d**) PVP + 10 CA (1000×), (**e**) PVP + 10 CA (500×).

Figure 1 presents SEM images of electrospun mats prepared from PVP in ethanol-aqueous (PVP), PVP in TE solvent (PVP/TE), PVP and PVP/TE containing 4 wt% CA, and PVP containing 10 wt% CA. Images (Figure 1) clearly show that uniform nano-microfiber mats were formed. The PVP fibers were randomly oriented and exhibited bead-free morphology (Figure 1a–d). According to Z. Czibulya and coauthors [31], the incorporation of 6.1 wt% CA into water-based PVA solutions did not significantly affect the structure of electrospun PVA mats. In contrast, in our study, only the addition of 10 wt% CA resulted in the formation of a porous membrane (Figure 1e); however, these membranes were brittle and were therefore excluded from further evaluation as packaging materials. It can be assumed that the ~38% increase in solution viscosity after CA addition (Table 1) reduced the mobility and mechanical flexibility, ultimately contributing to the formation of brittle PVP electrospun mats.

**Figure 2:**
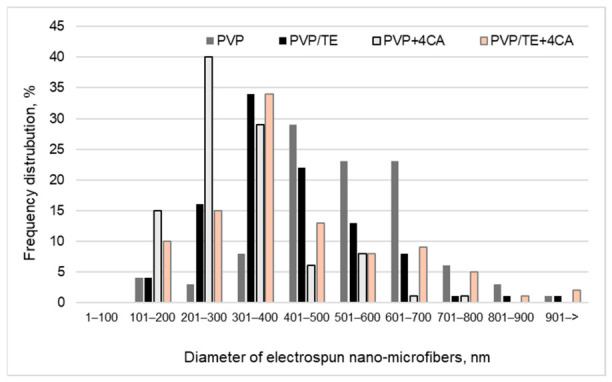
Diameter frequency distribution of PVP-based electrospun nano-microfibers.

The data in Figure 2 demonstrate that electrospinning of PVP in TE solution, or the incorporation of CA into the electrospinning solution, resulted in the formation of finer nano-microfibers. Electrospun fibers within the 1–300 nm diameter range were 7%, 20%, 55%, and 35% for PVP, PVP/TE, PVP + 4 CA, and PVP/TE + 4 CA formulations, respectively. Increasing the electrical conductivity of the solution generally leads to the formation of finer fibers with reduced bead formation [30]. Commonly, viscosity, influenced by polymer concentration and solvent properties, affects fiber thickness. High viscosity leads to thicker fibers, while low viscosity may result in bead-like structures [32]. According to the results presented in Figure 2, increase in electrospun solution conductivity and viscosity causes a decrease in fiber diameter. The conductivity values of the PVP + 4 CA and PVP/TE + 4 CA solutions were 36.0 ± 0.9 µS/cm and 48.9 ± 3.1 µS/cm, respectively (Table 1), which initiated the formation of finer nano-microfibers. Moreover, the conductivity of the PVP/TE solution reached 32.4 ± 1.8 µS/cm, compared with only 1.4 ± 0.2 µS/cm for PVP. As a result, 20% of the fibers produced from PVP/TE were within the 1–300 nm diameter range, whereas only 7% of the fibers obtained from PVP met this interval. The incorporation of TE, CA increases solution conductivity and affects viscosity and, presumably, molecular interactions. These changes intensify jet stretching during electrospinning, leading to finer, uniform nano-microfiber formation. Thus, TE and CA behave as a conductivity enhancer of the electrospinning solution, due to which thinner nano-microfibers were formed.

### 3.3. Optical Analysis of Electrospun Mats

**Figure 3:**
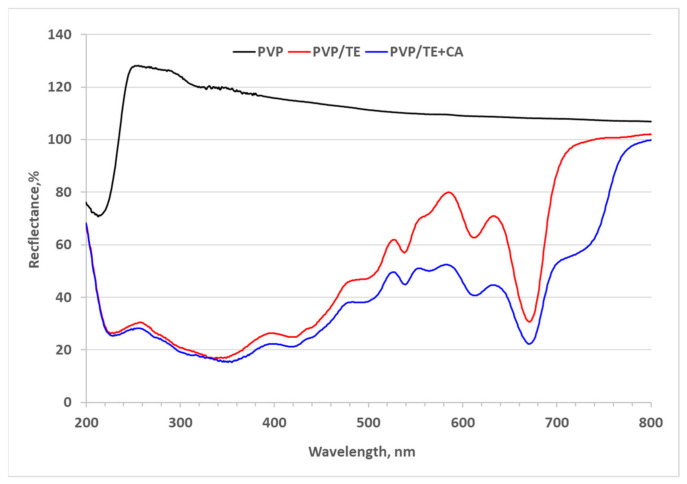
UV–VIS spectra of electrospun mats: PVP, PVP/TE, and PVP/TE + 4 CA.

Electrospun mats UV-VIS spectroscopy analysis was used to indicate how the light reflectance of the PVP mat changes with the addition of TE and CA. The curve in Figure 3 shows that light reflectance of pure PVP mat has a high light reflectance (even slightly higher than the PTFE-based diffuse reflectance standard Spectralon^®^, which is attributed to 100% reflectance [Spectralon^®^ Diffuse Reflectance Standards https://www.labsphere.com/] (accessed on 25 February 2026) in the entire visible electromagnetic radiation range (from 800 to 400 nm), including the entire near ultraviolet (from 400–300 nm) range and part of the middle ultraviolet range (from 300 to 235 nm) according ISO 21348 standard [33].

Only in the remaining narrow middle ultraviolet range (from 235 to 200 nm) is the PVP mat characterized by a decrease in radiation reflectance, i.e., increased absorption of UV radiation. Meanwhile, the addition of both TE and CA additives to the PVP mat leads to a very significant decrease in diffuse reflectance (or increase in absorbance) of visible and ultraviolet electromagnetic radiation. Since TE contains various bioactive compounds, some of which are polyphenolic pigment flavonoids, as well as a small concentration level of carotenoids, they can act as a natural colorant [34]; therefore, PVP with TE additive exhibits relatively higher diffuse reflectance intensity in the spectra around 450–650 nm, and at the end of the visible spectrum in the range from 700 to 800 nm, which corresponds to the yellow-to-orange and red-to-brownish hues of the PVP/TE and PVP/TE + CA samples. Meanwhile, the significant decrease in diffuse reflectance or increase in absorbance of electromagnetic radiation in the entire near-ultraviolet and part of the middle-ultraviolet range is most likely related to the characteristic spectral properties of phenolic derivatives. These compounds are usually colorless and have increased radiation absorption mainly in the UV region, and act as color stabilizers of other pigments and as co-pigments with anthocyanins, strengthening their color intensity [35,36].

### 3.4. FTIR Spectroscopy Electrospun Mats

**Figure 4:**
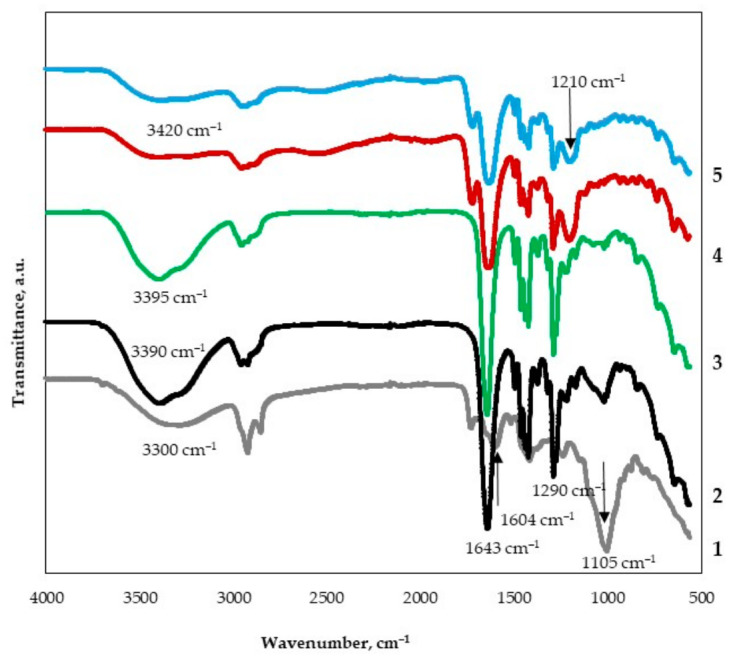
FTIR spectra for TE (1) and PVP electrospun mats: 2—pure PVP; 3—PVP/TE; 4—PVP + CA; 5—PVP/TE + CA.

FTIR spectroscopy was used to analyze the changes occurring in the PVP-based mats due to the presence of TE and CA (Figure 4). The spectrum of TE exhibits characteristic absorption bands that are consistent with phenolic-rich plant extracts and correlate well with the data reported by H. Maleki et al. [17]. In particular, the peaks observed at approximately 1005 cm^−1^, 1604 cm^−1^, and 3300 cm^−1^ are attributed to C–O stretching, C=O stretching, and –OH stretching vibrations, respectively (Figure 4, spectra 1). These bands are commonly associated with the major phenolic constituents of thyme, such as thymol and carvacrol. Electrospun PVP (Figure 4, spectra 2) mat spectrum displays its typical characteristic bands at 3390 cm^−1^ assigned to O–H stretching vibrations (associated with adsorbed moisture), and 2920 cm^−1^ corresponding to CH stretching vibrations [37]. Additional bands are observed at 1643 cm^−1^ (C=O stretching of the pyrrolidone ring), 1495 cm^−1^ and 1290 cm^−1^ (C-N stretching vibrations), as well as 1423 cm^−1^ attributed to C–H bending vibrations, confirming the structural integrity of PVP.

The obtained spectrum of the electrospun PVP/TE mats clearly shows the coexistence of characteristic absorption bands from both components (Figure 4, spectra 3 and 5). Minor shifts of several peaks to slightly higher or lower wavenumbers and partial band overlaps are observed, which can be attributed to physical intermolecular interactions, primarily hydrogen bonding, between the phenolic –OH groups of TE and the carbonyl groups of PVP [38]. Similar peak shifts and overlaps have been reported previously for the PVA–essential oil system [17], supporting this interpretation. The addition of CA introduces further spectral changes. CA exhibits a characteristic absorption peak near 1210^−1^, typically assigned to C–O stretching vibrations, which can serve as an indicator of its presence within electrospun mats (Figure 4, spectra 4 and 5). Furthermore, the broad O–H stretching band around 3420 cm^−1^ shifts to a lower wavenumber in CA-containing samples. This shift provides strong evidence of hydrogen-bond formation between CA, PVP, and the phenolic –OH groups of TE. These stronger hydrogen-bond-mediated associations reduce polymer chain mobility, which can modify the structural and mechanical properties of electrospun mats, leading to increased stiffness and, consequently, greater brittleness.

### 3.5. Application of Electrospun Mats as Active Fruit Packaging

**Figure 5:**
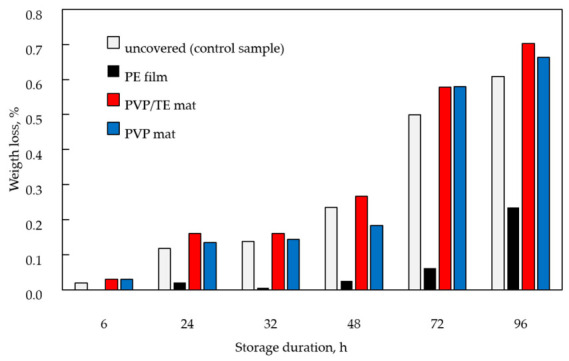
Weight loss of glass containers loaded with blueberries stored under different storage conditions.

**Figure 6:**
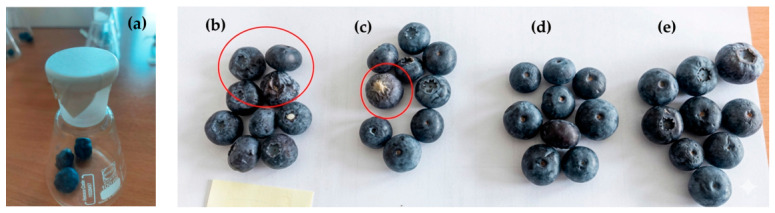
Experimental setup and visual appearance of blueberries after 96 h of storage: (**a**) glass container used for testing; (**b**) blueberries that were uncovered (control sample), (**c**) covered with PE film, (**d**) covered with PVP/TE mat, and (**e**) covered with PVP mat. Red circles show damaged blueberries.

The effectiveness of electrospun mats as an active packaging film was evaluated using blueberries. Blueberries are widely recognized as one of the most nutritionally valuable fruits globally and are frequently classified as a “superfruit” due to their distinctive sensory properties and exceptionally high concentrations of health-promoting bioactive compounds [39]. They contain significant amounts of organic acids, phenolic constituents, sugars, minerals, vitamins, dietary fiber, and pectin, all of which contribute to their considerable nutritional and functional value for human health. Despite these benefits, fresh blueberries exhibit high perishability and are particularly vulnerable to mechanical damage and microbial contamination, factors that markedly limit their postharvest stability and shelf life. PLA electrospun mats loaded with hydroxypropyltrimethyl ammonium chloride chitosan and anthocyanins, poly(lactic-co-caprolactone)/gelatin mats cross-linked with tannic acid, and K-carrageenan/PLA mats loaded with vitamin C (VC)/2-hydroxypropyl-β-cyclodextrin have been used by other researchers to monitor blueberry freshness and extend shelf life [39,40,41]. In this study, the effect of PVP-based electrospun mats on blueberry weight loss and visual quality was investigated, and the obtained results are presented in Figure 5 and Figure 6.

As can be seen from Figure 5, the weight loss of blueberries gradually increased with storage time. The slowest weight loss was observed in samples covered with food-grade PE wrapping film (0.23% after 96 h). This can be attributed to the nonporous nature of PE film and its low water vapor transmission rate, which promotes the accumulation of humidity inside the glass container. Such conditions, while reducing weight loss, increase the risk of spoilage, as evidenced by the visual appearance of the blueberries (Figure 6c). In contrast, electrospun PVP and PVP/TE mats are porous and breathable, which facilitates the diffusion of water vapor out of the container. As a result, moisture is continuously released to the surrounding environment, leading to a markedly higher weight loss (PVP—0.66%, PVP/TE—0.7%, after 96 h) compared with samples covered with the impermeable PE film. However, unlike uncovered samples, the electrospun mats act as humidity buffers, maintaining a more stable and moderately humid microclimate directly around the berry surface. It is possible to make an assumption that this localized humidity reduces surface dehydration rates and mechanical stresses associated with rapid and uneven drying in deformable porous materials. Consequently, although water is lost from the system as a whole (as reflected by weight loss), the berries do not undergo severe structural collapse, resulting in limited shrinkage and reduced wrinkling (Figure 6d,e). Importantly, shrinkage is primarily linked to mechanical failure induced by rapid, non-uniform moisture loss at the surface, whereas gradual and uniform moisture exchange can mitigate this effect. Therefore, weight loss and shrinkage are not necessarily directly proportional. Similar results were reported in He et al.’s study [42], where cross-linked electrospun PVA/CS/tannic acid mats incorporating ZnO showed excellent moisture retention properties that prevented dehydration and shrinkage of strawberries.

Table 2 presents quantitative analysis of blueberry preservation corresponding to Figure 6. The quantitative data confirms that the uncovered berries underwent significant moisture loss, losing approximately 31% of their surface area (SI = 0.69) and exhibiting high wrinkling (WAI = 0.82). In contrast, blueberries covered with PVP electrospun mats showed the highest preservation efficiency. Specifically, blueberries covered with the PVP/TE electrospun mat maintained high turgor and a smooth surface morphology, preserving 96% of their original surface area and exhibiting a significantly lower wrinkle index (WAI = 0.14). Blueberries covered with PE film showed only minor and localized shriveling, suggesting partial moisture retention; however, visible surface irregularities still reflected progressive moisture loss.

In summary, PVP-based electrospun mats allow efficient vapor transport while simultaneously protecting the fruit from rapid surface dehydration, leading to a combination of high weight loss but better preservation of shape and surface integrity.

**Table 2:** Quantitative analysis of blueberry preservation.

Blueberry Sample	Storage Conditions in Glass Container	WAI (0 to 1 Scale)	SI (Relative Area)
(b) control	Uncovered	0.82 ± 0.07	0.69 ± 0.05
(c)	PE film covered	0.35 ± 0.04	0.88 ± 0.03
(d)	PVP/TE mat covered	0.14 ± 0.02	0.96 ± 0.02
(e)	PVP mat covered	0.18 ± 0.03	0.94 ± 0.02

## 4. Conclusions

In this study, electrospun PVP nano-microfiber mats containing *T. vulgaris* extract and citric acid were successfully fabricated as an advanced, sustainable, and fully water-soluble material with strong potential for active food packaging applications. The presence of *T. vulgaris* and citric acid in the PVP solution enhanced its viscosity and electrical conductivity, thereby facilitating the formation of electrospun mats with finer nano-microfibrous structures. These changes are attributed to the establishment of hydrogen-bonded networks and strengthened intermolecular interactions among PVP chains, citric acid, and bioactive constituents of the extract. The storage results demonstrate that PVP-based electrospun mats are a promising active packaging option for fresh blueberries. Although these breathable mats led to higher overall weight loss than impermeable food-grade PE film due to enhanced moisture diffusion, they created a more stable and moderately humid microenvironment at the fruit surface. This controlled moisture exchange reduced tissue collapse, shrinkage, and wrinkling, resulting in better visual quality during storage. Therefore, weight loss was not directly correlated with visual deterioration, highlighting the advantage of electrospun mats as humidity buffering systems for maintaining blueberry appearance and structural integrity. Collectively, these findings highlight the strong potential of electrospun PVP/*T. vulgaris* extract mats as an effective, functional, and environmentally friendly next-generation active packaging system.

## Figures and Tables

**Table 1:** Viscosity and conductivity of electrospun solutions.

Code	Viscosity, mPa·s ± Δ	Conductivity, µS/cm ± Δ
PVP	560.0 ± 16.2	1.4 ± 0.2
PVP/TE	844.4 ± 28.5	32.4 ± 1.8
PVP + 4 CA	830.8 ± 27.5	36.0 ± 0.9
PVP/TE + 4 CA	865.4 ± 41.9	48.9 ± 3.1
PVP + 10 CA	912.0 ± 25.8	35.9 ± 7.2
PVP/TE + 10 CA	1218.0 ± 45.4	57.7 ± 8.3

## Data Availability

The original contributions presented in this study are included in the article. Further inquiries can be directed to the corresponding author.
